# Prolonged Nitric Oxide Exposure Enhances Anoikis Resistance and Migration through Epithelial-Mesenchymal Transition and Caveolin-1 Upregulation

**DOI:** 10.1155/2014/941359

**Published:** 2014-05-20

**Authors:** Pithi Chanvorachote, Varisa Pongrakhananon, Preedakorn Chunhacha

**Affiliations:** Department of Pharmacology and Physiology, Faculty of Pharmaceutical Sciences and Cell-Based Drug and Health Products Development Research Unit, Chulalongkorn University, Pathumwan, Bangkok 10330, Thailand

## Abstract

Nitric oxide (NO) in tumor microenvironment may have a significant impact on metastatic behaviors of cancer. Noncytotoxic doses of NO enhanced anoikis resistance and migration in lung cancer H23 cells via an increase in lamellipodia, epithelial-mesenchymal transition (EMT) markers including vimentin and snail, and caveolin-1 (Cav-1). However, the induction of EMT was found in Cav-1-knock down cells treated with NO, suggesting that EMT was through Cav-1-independent pathway. These effects of NO were consistently observed in other lung cancer cells including H292 and H460 cells. These findings highlight the novel role of NO on EMT and metastatic behaviors of cancer cells.

## 1. Introduction

Lung cancer is among the leading causes of cancer-related death worldwide and evidences have suggested that metastasis in such a cancer is a major cause of death [1]. As metastasis is a complicated process, cancer cells must have an ability to overcome several obstacles including anoikis, a process of death mediated after cells detachment [2]. Anoikis is accepted as one important body defense mechanism against cancer dissemination [2]. Like adherent normal cells, most solid tumor cells will die after detachment by anoikis; however, certain population of the cells have a capability to resist anoikis, survive in the blood or lymphatic circulations, reach new sites, and establish secondary tumors. Besides anoikis resistance, a motility behavior of cancer cells was also recognized as a critical factor for success in metastasis as the early step of cancer dissemination involves cell migration and intravasation into blood or lymphatic systems [3]. A number of studies in the cancer research fields have focused on the biological process found in cancer cells called epithelial-mesenchymal transition (EMT) and EMT is believed to enhance metastatic potentials of several cancers [4]. Indeed, EMT is a multistep cellular process that allows an epithelial cell to possess mesenchymal phenotype [5]. Recently, EMT has garnered special attention since many researchers recognized EMT as a hallmark reflecting cancer aggressiveness and poor prognosis [6]. An enhanced metastatic behavior such as an increase in migratory activity was continuously demonstrated in cancer cells exhibiting EMT phenotype [5, 6]. Also, the EMT was shown to be involved with anoikis resistance in lung, melanoma and colon cancer cells [7–9]. Downregulation of E-cadherin, together with upregulation of N-cadherin, vimentin, and snail, was long shown to be a key indicator of EMT process; therefore, the protein alterations were shown to link with the acquisition of anoikis resistance [6, 10–12]. Likewise, caveolin-1 (Cav-1), a major protein component of caveolae, was reported to regulate cancer cell activities. Caveolin-1 expression in lung cancer was shown to be related to poor prognosis and metastasis capability [13]. Our previous study showed that Cav-1 mediated anoikis resistant [14, 15] as well as increased migration and invasion in lung cancer cells [16]. Together, such information leads to the possible conclusion that EMT and Cav-1 may share overlapping pathways in regulation of metastatic behaviors; however, insights into such regulation remain elusive.

Nitric oxide (NO) is a gaseous biological mediator that frequently reported to be upregulated in lung cancer environments [17]. Our previous works demonstrated that this important mediator affects lung cancer cells in many ways including induced cisplatin [18] and Fas ligand resistance [19]. However, its roles in regulation of EMT remain unknown.

So far, the knowledge regarding the biological mediators that force EMT in lung cancer has been largely unknown. Because more understanding of nature of the cancer cells in response to biological substance may lead to high precision and efficiency in treating the disease, the present study aimed to investigate an effect of long-term NO exposure on EMT characteristics and Cav-1 level in lung cancer cells on the basis that the results gained from the study could benefit the development of therapeutic approaches.

## 2. Materials and Methods

### 2.1. Cells and Reagents

The non-small cell lung cancer (NSCLC) cell lines H23, H292, A549, and H460 were obtained from the American Type Culture Collection (Manassas, VA). The cells were cultured in RPMI 1640 supplemented with 5% fetal bovine serum (FBS), 2 mM L-glutamine, and 100 units/mL penicillin/streptomycin. The cells were incubated in a 5% CO2 environment at 37°C. For NO exposure, cells were cultured in medium containing DPTA NONOate (at nontoxic concentrations) for 14 days. The culturing medium was replaced by freshly prepared medium containing DPTA NONOate every 2 days. Phalloidin tetramethylrhodamine B isothiocyanate, dipropylenetriamine NONOate (DPTA NONOate), and 3-(4,5-dimethylthiazol-2-yl)-2,5-diphenyltetrazolium bromide (MTT) were obtained from Sigma Chemical, Inc. (St. Louis, MO, USA). Hoechst 33342 was obtained from Molecular Probes, Inc. (Eugene, OR). Antibodies for caveolin-1, vimentin, snail, TCF8/ZEB1, E-cadherin, ZO-1, N-cadherin, *β*-catenin, and *β*-actin, as well as peroxidase-conjugated secondary antibodies were obtained from Cell Signaling Technology, Inc. (Danvers, MA).

### 2.2. Cell Viability Assay

Cell viability was determined by the MTT assay. After specific treatments, cells were incubated with 500 *μ*g/mL of MTT at 37°C for 4 h. Then, the MTT solution was removed and 100 *μ*L of DMSO was added to dissolve the formazan crystal. The intensity reading of MTT product was measured at 570 nm using a microplate reader, and the percentage of viable cells was calculated in relation to control cells.

### 2.3. Cell Morphology and Lamellipodia Characterization

Cell morphology and lamellipodia were investigated by a phalloidin-rhodamine staining assay. The cells were washed with PBS, fixed with 4% paraformaldehyde in PBS for 10 min at 37°C, permeabilized with 0.1% Triton-X100 in PBS for 4 min, and blocked with 0.2% BSA for 30 min. Then, the cells were incubated with either 1 : 100 phalloidin-rhodamine in PBS or 0.4% sulforhodamine B in 1% acetic acid for 15 min, rinsed 3 times with PBS, and mounted with 50% glycerol. Cell morphology was then assessed by fluorescent imaging (Olympus IX51 with DP70). Filopodia protrusion was represented as the average number of filopodia/cell relative to untreated cells in each field.

### 2.4. Anoikis Assay

For anoikis evaluation, 6-well tissue culture plates were coated with 200 *μ*L of poly 2-hydroxyethylmethacrylate (poly-HEMA; Sigma) and left for 10 h in a laminar flow hood. Cells were seeded in poly-HEMA-coated plates at the density of 1 × 10^5^ cells/mL and incubated for various times up to 24 h at 37°C. Cell viability was assessed by addition of 1 : 50 resazurin for 1 h at 37°C. Fluorescence intensity of resazurin product (resorufin) was measured at 530 nm (excitation wavelength) and 590 nm (emission wavelength) using a microplate reader. Cell viability was calculated as a percentage relatively to time zero. All analyses were performed in at least three independent replicate experiments.

### 2.5. Migration Assay

The migration assay was carried out using Transwell cell culture chamber (Corning Costar number 3422, MA, USA). Conditioned media (500 *μ*L media with 10% FBS) were added into the lower compartment of the chamber. P1, A549, H23, and H460 cells at the concentration of 2 × 10^5^ cells in 1% fetal bovine serum containing media were added to the upper compartment of the chamber. After 12 h incubation, the top side of insert membrane was scrubbed with a cotton swab and the bottom side was fixed with ice-cold methanol and stained with Hoechst 33342 and scoring under fluorescence microscope (Olympus IX51 with DP70).

### 2.6. Plasmid and Transfection

Cav-1 knockdown plasmid short hairpin (sh)RNA-Cav-1 was obtained from Santa Cruz Biotechnology. Stable transfection of Cav-1 knockdown plasmid was generated by culturing cells in a six-well plate until they reached 60% confluence. Fifteen *μ*L of lipofectamine reagent and 2 *μ*g of Cav-1, shRNA-Cav-1, or control plasmid were used to transfect the cells in the absence of serum. After 12 h the medium was replaced with culture medium containing 5% FBS. Approximately 36 h after the beginning of transfection, the cells were digested with 0.03% trypsin, and the cell suspensions were plated onto 75-mL culture flasks and cultured for 24 to 28 days with specific selection. The stable transfectants were pooled, and the expression of Cav-1 protein in the transfectants was confirmed by western blotting. The cells were cultured in antibiotic-free RPMI 1640 medium for at least two passages before they were used in each experiment.

### 2.7. Western Blotting

After specific treatments, cells were incubated in lysis buffer containing 20 mM Tris-HCl (pH 7.5), 1% Triton X-100, 150 mM sodium chloride, 10% glycerol, 1 mM sodium orthovanadate, 50 mM sodium fluoride, 100 mM phenylmethylsulfonyl fluoride, and a commercial protease inhibitor cocktail (Roche Molecular Biochemicals, Basel, Switzerland) for 30 min on ice. Cell lysates were collected and determined for protein content using the Bradford method (Bio-Rad Laboratories, Hercules, CA). An equal amount of proteins of each sample (40 *μ*g) were denatured by heating at 95°C for 5 min with Laemmli loading buffer and subsequently loaded on 10% SDS-polyacrylamide gel electrophoresis. After separation, proteins were transferred onto 0.45 *μ*m nitrocellulose membranes (Bio-Rad). The transferred membranes were blocked for 1 hour in 5% nonfat dry milk in TBST (25 mM Tris-HCl (pH 7.5), 125 mM NaCl, 0.05% Tween-20) and incubated with the appropriate primary antibodies at 4°C overnight. Membranes were washed twice with TBST for 10 min and incubated with horseradish peroxidase-coupled isotype-specific secondary antibodies for 1 h at room temperature. The immune complexes were detected by enhancement with chemiluminescence substrate (Supersignal West Pico; Pierce, Rockford, IL) and quantified using analyst/PC densitometry software (Bio-Rad).

### 2.8. Statistical Analysis

The data represented means ± SD from three or more independent experiments. Statistical analysis was performed by Student's *t*-test at a significance level of *P* < 0.05.

## 3. Results

### 3.1. Nitric Oxide Exposure Alters Cell Morphology of Human Non-Small Cell Lung Cancer H23 Cells

**Figure 1:**
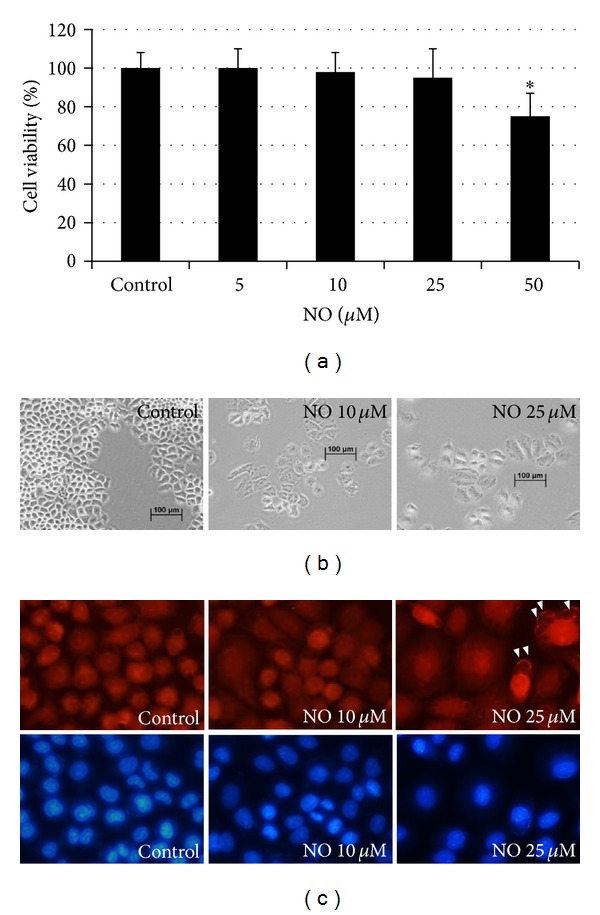
Effect of nitric oxide donor on cytotoxicity in lung carcinoma H23 cells. (a) Effect of DPTA NONOate on cell viability. Lung cancer H23 cells were treated with various concentrations (0–50 *μ*M) of DPTA NONOate for 24 h. The cell viability was analyzed using the MTT assay. The data are the mean ± S.D. (*n* = 3). *P < 0.05 versus the nontreated control. (b) Morphology of H23 cells treated with DETA NONOate for 14 days. (c) Lamellipodia formation in H23 cells treated with NO donor. H23 cells were treated with NO donor at concentrations of 10 and 25 *μ*M for 14 days. The cells were then stained with phalloidin-rhodamine and Hoechst33342 dye.

To elucidate the effect of NO, a mediator frequently found in tumor microenvironments on metastatic potentials, we first characterized NO donor that causes no toxicity to the cells. H23 cells were incubated with DETA NONOate for 72 h and viability of the cells was determined by MTT-based viability assay.  Figure 1(a) shows that the treatment with NO donor (DETA NONOate) at the concentrations ranging from 0–25 *μ*M caused no significant cytotoxic effect on H23 cell viability in comparison to nontreated control. For further investigations, cells were cultured in the normal growth medium supplemented with NO donor at the concentrations of 10 and 25 *μ*M for 14 days. Interestingly, NO-treated cells exhibited dramatically change in terms of cell morphology with cell enlargement in comparison to that of control nontreated cells (Figure 1(b)).

Lamellipodia are a cell protrusion that is widely accepted to be critical for directional migration in many cells [20]. To clarify whether such cell enlargement is caused by an increase of lamellipodia, we performed phalloidin-rhodamine staining assay with Hoechst 33342 nuclear staining.  Figure 1(c) indicates that NO-treated cells exhibited increased sheet-like lamellipodia in comparison to those of parental H23 cells. Since lamellipodia were shown to increase in highly motile cells, these results suggested that long-term NO exposure may result in an increase of cell migratory activity.

### 3.2. Nitric Oxide Exposure Induces Cell Migration and Anoikis Resistance

**Figure 2:**
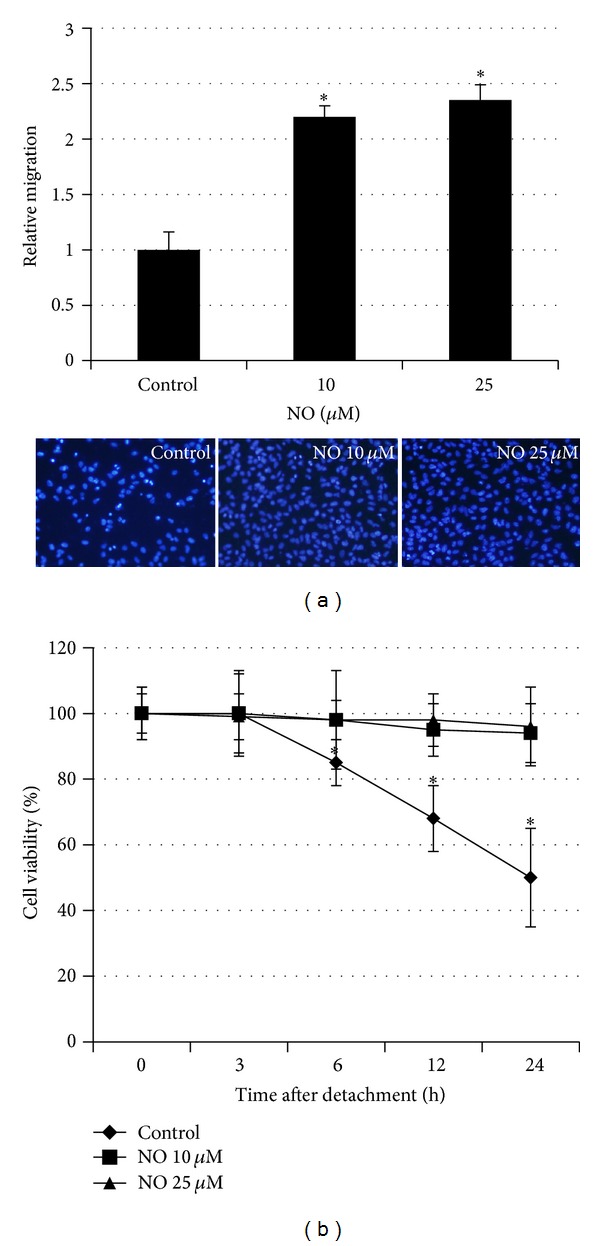
Effect of nitric oxide exposure on cell migration and anoikis response. Cells were exposed to DETA NONOate at various concentrations (10 and 25 *μ*M) for 14 days and subjected to migration and anoikis assays. (a) The relative cell migration was determined as described in Material and Methods Section. (b) Viability of the cells after detachment was evaluated in a time-dependent manner. The data are the mean ± S.D. (*n* = 3). *P < 0.05 versus the control cells.

Having shown that NO treatment has an inductive effect on cell protrusion, we next characterized migratory activity of the cells. NO-treated (14 days) and parental H23 cells were subjected to migration assay as described in Materials and Methods Section.  Figure 2 shows that lung cancer cells exposed to NO for 14 days exhibited significantly enhanced migration activity. Even though the dose-dependent effect of NO in this case was not observed, an increase in cell migration approximately 2 folds in both NO-treated conditions strongly indicates potentiating role of NO on motility of these cancer cells. Since migration and anoikis resistance are accepted to be important factors for metastasis, we next elucidated the effect of NO exposure on anoikis susceptibility of the cells. Cells were similarly treated with NO as the aforementioned for 14 days and were determined for anoikis sensitivity by anoikis assay.  Figure 2(b) indicates that the NO-treated cells at concentrations of 10 and 25 *μ*M exhibited anoikis resistant phenotype and could survive in detached condition up to 24 h, whereas their nontreated counterparts showed 50% reduction in cell viability after detachment for 24 h. Together, these results suggested that long-term exposure of the cells to NO potentiates anoikis resistance as well as migration ability of H23 cells and may be responsible for the metastasis potentials.

### 3.3. NO Induces Epithelial to Mesenchymal Transition

**Figure 3:**
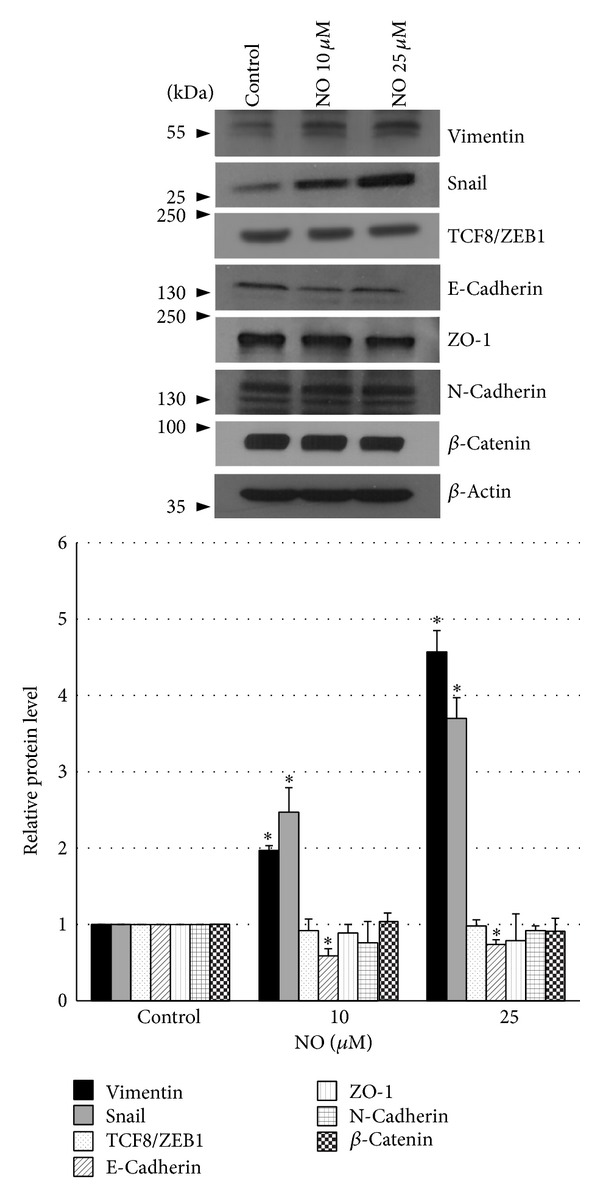
Effect of nitric oxide on epithelial-mesenchymal transition. Cells were exposed to DETA NONOate at various concentrations (10 and 25 *μ*M) for 14 days and subjected to migration and anoikis assays. The expression level of EMT markers including vimentin, snail, TCF8/ZEB1, E-cadherin, ZO-1, N-cadherin, and *β*-catenin were determined using western blot analysis. To confirm equal loading of the samples, the blots were reprobed with *β*-actin antibody. The immunoblot signals were quantified by densitometry. The data are the mean ± S.D. (*n* = 3). *P < 0.05 versus the nontreated control.

Enhanced abilities of cancer cells to metastasis are believed to increase through the process of EMT. Also, EMT was linked to the increasing capability of cancer cells in migrating away and resisting anoikis [9]. The present study further investigated the EMT phenotypes of the long-term NO-treated and H23 cells. The expression levels of EMT markers including vimentin, snail, TCF8/ZEB1, E-cadherin, ZO-1, N-cadherin, and *β*-catenin were determined using western blot analysis. The cells were exposed to NO donor at the concentrations of 10 and 25 *μ*M for 14 days and EMT markers were evaluated.  Figure 3 shows that NO treatment significantly decreased E-cadherin level in the cells. Interestingly, vimentin and snail dramatically upregulated in NO-treated H23 cells in a dose-dependent manner, while other markers TCF8/ZEB1, ZO-1, N-cadherin, and *β*-catenin were not significantly altered. We found that at the concentration of 10 µM, NO donor induced approximately 2- and 2.5-fold inductions of vimentin and snail, respectively. Moreover, at 25 *μ*M, NO donor induced approximately 4.5 and 4 fold inductions of vimentin and snail, respectively.

Collectively, our results indicated that the long-term treatment of NO induced EMT in lung cancer H23 cells and such EMT may be responsible for the increase of migration and anoikis resistance.

### 3.4. NO Treatment Increases Caveolin-1 Level

**Figure 4:**
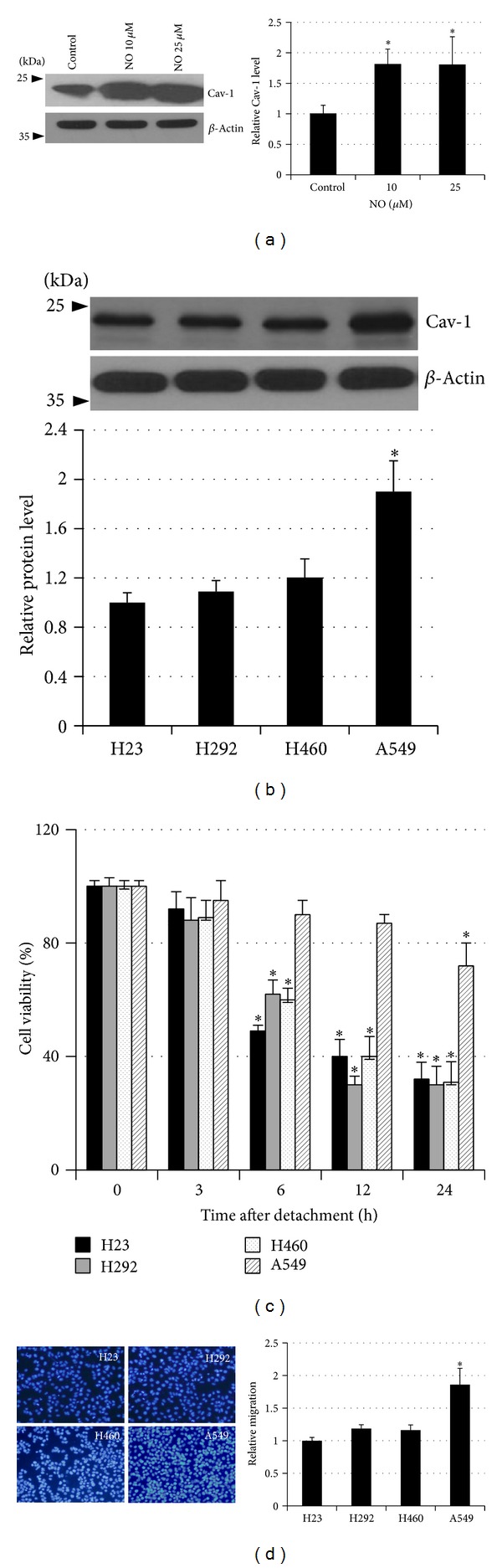
Nitric oxide mediated caveolin-1 upregulation, anoikis resistance and increase migration. (a) Cells were exposed to DETA NONOate for 14 days and Cav-1 level was evaluated by western blotting. (b) Basal level of Cav-1 protein in H23, H292, H460, and A549 lung cancer cells was determined and the immunoblot signals were quantified by densitometry. (c) Anoikis susceptibility of H23, H292, H460, and A549 lung cancer cells was determined by anoikis assay. (d) The relative cell migration of H23, H292, H460, and A549 lung cancer cells was determined. The data are the mean ± S.D. (*n* = 3). *P < 0.05 versus the H23 control cells.

We and others have provided a number of evidences indicating that Cav-1 protein performs a key function in regulation of anoikis resistance as well as cell motility [14–16]. These data lead to the possibility that long-term NO exposure could mediate such cancer cell aggressiveness through Cav-1-dependent mechanism. The parental and NO-treated cells (14 days) were subjected to western blot analysis for Cav-1 determination. The results indicated that after the treatment with NO for 14 days, Cav-1 in H23 cells was significantly upregulated (Figure 4(a)). Further, we provided the evidence demonstrating the roles of Cav-1 on anoikis resistance and cell migration in H23 and other lung cancer cells. Basal Cav-1 level of H23, H292, H460, and A549 was evaluated using western blotting (Figure 4(b)). Further, the anoikis susceptibility and cell migration of all cells were determined. A549 cells exhibiting the highest level of Cav-1 showed the strongest anoikis resistant potential in comparison to that of H23, H292, and H460 lung cancer cells, suggesting the role of Cav-1 in attenuating anoikis process in these cells (Figure 4(c)). Likewise, the level of Cav-1 protein was found to be tightly correlated with migratory activity of the cells (Figure 4(d)). Taken together, our results show that long-term NO exposure increased Cav-1 level in H23 cells and the protein, at least partly, mediated anoikis resistance and increased cell motility.

### 3.5. NO Induces EMT in a Cav-1-Independent Mechanism

**Figure 5:**
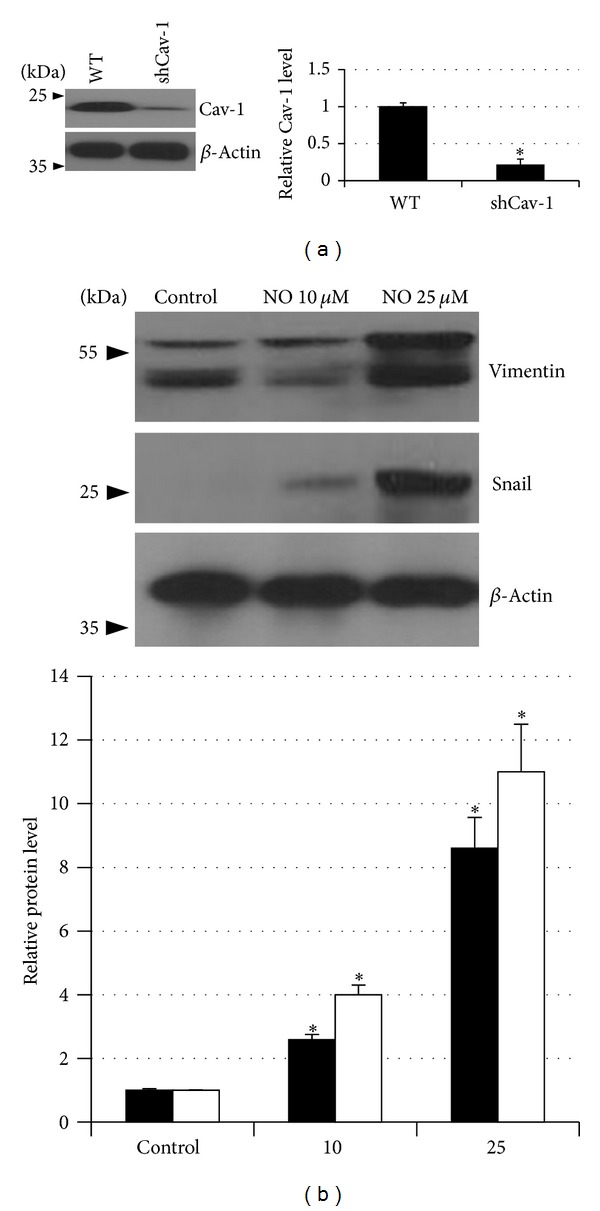
Nitric oxide mediated epithelial-mesenchymal transition via Cav-1-independent mechanism.Stable Cav-1 knock-down (ShCav-1) cells were established as indicated in Section 2. (a) The expression level of Cav-1 protein in the control H23 and ShCav-1 cells was determined by western blotting. (b) The ShRNA-Cav-1 cells were exposed to NO donor for 14 days, and the expression of vimentin and snail was determined. The immunoblot signals of vimentin and snail were quantified by densitometry. The data are the mean ± S.D. (*n* = 3). *P < 0.05 versus the nontreated control.

To test NO-mediated EMT in these cells through Cav-1-dependent pathway, H23 cells were stably transfected with ShRNA-Cav-1 plasmid. The stable Cav-1 knock-down cells were tested for the Cav-1 level, and the results indicated that Cav-1 in the cells dramatically decreased (Figure 5(a)). Then, the ShRNA-Cav-1 cells were treated with NO donor as described previously, and EMT markers were evaluated. Even though Cav-1 in the cells was suppressed, vimentin and snail were significantly upregulated in response to long-term NO treatment (Figure 5(b)). These results indicated that the EMT process induced by NO exposure in our system may not mediate through Cav-1-dependent mechanism.

### 3.6. NO Induces EMT in Other Lung Cancer Cells

**Figure 6:**
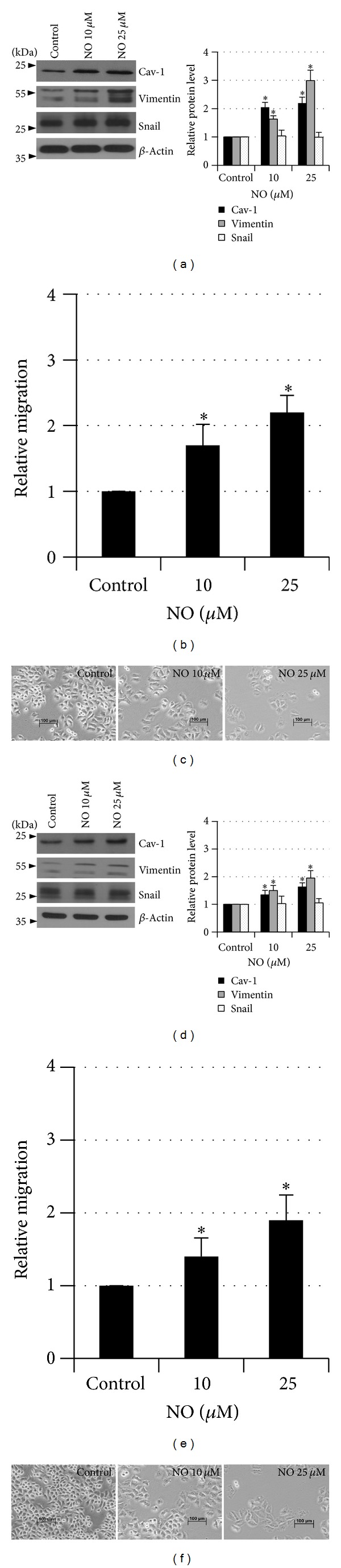
Nitric oxide mediated caveolin-1 upregulation and epithelial-mesenchymal transition in other lung cancer cells. H292 (a) and H460 (d) lung cancer cells were exposed to DETA NONOate for 14 days and expression levels of Cav-1, vimentin, and snail were determined. The immunoblot signals were quantified by densitometry. Migratory activity of H292 (b) and H460 (c) cells was evaluated as described. Morphology of H292 (c) and H460 (f) cells treated with DETA NONOate for 14 days. The data are the mean ± S.D. (*n* = 3). *P < 0.05 versus the nontreated control.

Further, we tested whether other lung cancer cells responded to NO exposure in the same way as H23 cells. Lung cancer H292 and H460 cells were exposed to NO at the concentrations of 10 and 25 *μ*M for 14 days and EMT markers and aggressive behaviors of the cells were investigated. Figures 6(a) and 6(d) indicate that H292 cells exhibited a significant increase in Cav-1 and vimentin levels in response to NO treatment, while snail level in these cells was not altered. These results suggested that long-term NO exposure resulted in an increase in both Cav-1 protein and EMT process. Consistently, the migratory activity of these cells significantly enhanced in the cells treated with NO. Taken together, our experiments indicated that long-term NO exposure was able to increase cellular Cav-1 protein and induced EMT.

## 4. Discussion

The knowledge regarding factors that influence cancer metastasis may benefit the development of novel treating strategy as well as the improved diagnosis sensitivity to this life-threatening disease. Nowadays, evidences suggest that metastasis process of cancers can be enhanced by several factors. Indeed, the increased levels of inflammatory cytokines, reactive oxygen species, and nitric oxide in cancer microenvironment may have an important impact on cell aggressiveness [17, 21, 22]. Recently, we have provided the data indicating that Cav-1 in the detached lung cancer cells could inhibit anoikis process of the cells by sustaining the antiapoptotic Mcl-1 protein [23]. Additionally, in terms of cancer cell migration, Cav-1 protein is shown to be a positive regulator [16]. Among several biological mediators, NO has received an increasing attention in the cancer research field and is believed to be the key factor potentiating cancer progression [21]. However, the evidence in the regulatory role of this substance on EMT and cancer aggressiveness is still limited. Herein, we provide the evidence indicating that the cells that received NO at nontoxic concentration for relatively long period made the cells more capable of spreading by increasing Cav-1 and EMT.

Current evidences explaining the involvement of cancer metastasis and the process of EMT and EMT are accepted to be a mechanism facilitating the cancer cells to spread away [4, 6, 24]. Indeed, the roles of NO on EMT process are intriguing and sometimes contradictory depending on cell type, duration of NO exposure, and dose of NO-modulating agents. Some previous studies suggested that NO attenuates TGF-*β*1-mediated EMT in alveolar epithelial cells [25] and mouse hepatocytes [26]. Also, NO was shown to suppress EMT in prostate cancer cells when it was used at high concentrations [27]. Based on the fact that EMT is a complex process which is involved in multiple signaling pathways, the distinguishable effect of NO reported by the present study may be due to the difference in type of cells as well as the concentrations of NO.

We demonstrated that after prolonged NO exposure, the lung cancer H23 cells increased the expression level of vimentin and snail in concomitant with the decrease of E-cadherin. In response to such EMT, the migratory as well as anoikis resistance characteristics in NO-treated cancer cells were enhanced (Figure 2). In addition, in other lung cancer cells the NO mediated EMT and increased metastatic behaviors could be observed (Figure 6). Such information has strengthened the link between NO and EMT process in lung cancer. Together with the fact that not all cancer cells exhibited high EMT [24], the cells that immerged in NO-rich environment may have a better chance to succeed metastasis through EMT enhancement.

Regarding Cav-1 protein, certain studies have focused on the role of Cav-1 expression in increasing migration and anoikis resistance [15, 16, 22, 23]. Although some evidence has suggested the role of Cav-1 in suppressing cancer [28], in lung cancer, Cav-1 potentiates cancer progression and aggressiveness. Cav-1 expression has been shown to relate to poor prognosis and reduced tumor-free periods in lung cancer patients [29]. Moreover, Cav-1 was shown to facilitate metastasis and induce anoikis resistance in lung carcinoma cell lines [2, 14–16, 30]. Not only does Cav-1 play a role in cell death and survival, but also in cell migration [16], invasion [31], and lipid transportation [32]. However, in our study, NO could induce EMT characteristics even in Cav-1 knock-down cells. This suggests that NO mediated EMT in Cav-1 independent mechanism. Cav-1 may possess an ability to overcome anoikis in the cells by direct interaction with Mcl-1 protein [22]. In addition, Cav-1 was shown to increase cellular level of activated Akt that may directly contribute to the increase cell survival as well as migration [16].

Based on these data, we have provided the novel information that the ability of cancer cells to transition to mesenchymal phenotype could be enhanced by long-term NO exposure. Additionally, NO exposure increases the level of Cav-1, a known protein facilitating anoikis resistance and migration. Our results support the conception that the biological mediators found in cancer microenvironments have a significant impact on the ability of cancer cells to metastasise. This insight may facilitate the better understanding of cancer cell biology.
